# Supplementary figures and images for: Microtubules restrict F-actin polymerization to the immune synapse via GEF-H1 to maintain polarity in lymphocytes
Source: eLife. 2022 Sep 16;11:e78330. doi: 10.7554/eLife.78330 (PMC9592083; doi:10.7554/eLife.78330)

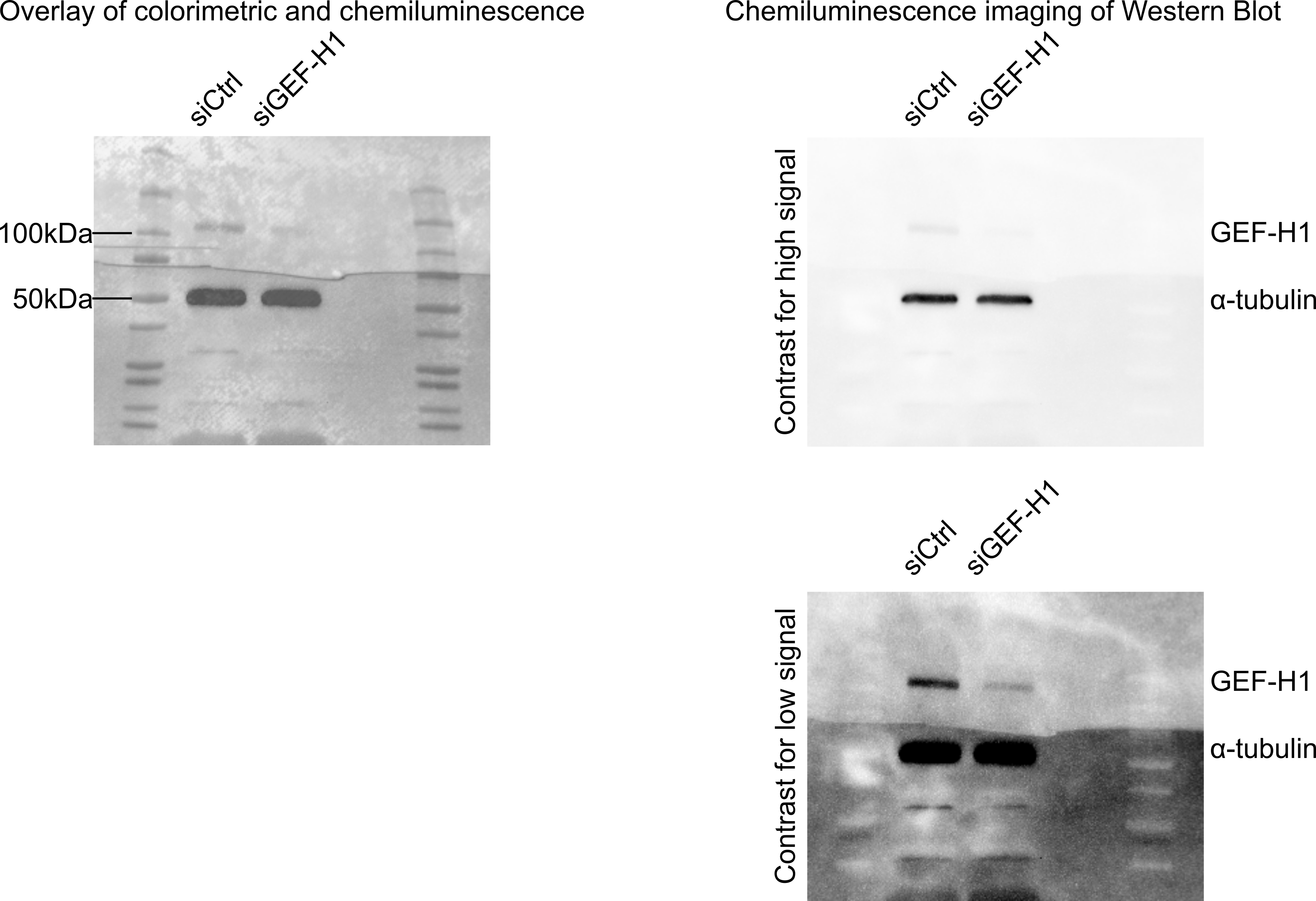

Supplement: Figure 6—source data 1. [file elife-78330-fig6-data1.zip › Figure6_SourceData1/Figure6_SourceData1_imagesfullgel.png]
